# Eyelid Opening with Trigeminal Proprioceptive Activation Regulates a Brainstem Arousal Mechanism

**DOI:** 10.1371/journal.pone.0134659

**Published:** 2015-08-05

**Authors:** Kiyoshi Matsuo, Ryokuya Ban, Yuki Hama, Shunsuke Yuzuriha

**Affiliations:** Department of Plastic and Reconstructive Surgery, Shinshu University School of Medicine, Matsumoto, Japan; Tokai University, JAPAN

## Abstract

Eyelid opening stretches mechanoreceptors in the supratarsal Müller muscle to activate the proprioceptive fiber supplied by the trigeminal mesencephalic nucleus. This proprioception induces reflex contractions of the slow-twitch fibers in the levator palpebrae superioris and frontalis muscles to sustain eyelid and eyebrow positions against gravity. The cell bodies of the trigeminal proprioceptive neurons in the mesencephalon potentially make gap-junctional connections with the locus coeruleus neurons. The locus coeruleus is implicated in arousal and autonomic function. Due to the relationship between arousal, ventromedial prefrontal cortex, and skin conductance, we assessed whether upgaze with trigeminal proprioceptive evocation activates sympathetically innervated sweat glands and the ventromedial prefrontal cortex. Specifically, we examined whether 60° upgaze induces palmar sweating and hemodynamic changes in the prefrontal cortex in 16 subjects. Sweating was monitored using a thumb-mounted perspiration meter, and prefrontal cortex activity was measured with 45-channel, functional near-infrared spectroscopy (fNIRS) and 2-channel NIRS at Fp1 and Fp2. In 16 subjects, palmar sweating was induced by upgaze and decreased in response to downgaze. Upgaze activated the ventromedial prefrontal cortex with an accumulation of integrated concentration changes in deoxyhemoglobin, oxyhemoglobin, and total hemoglobin levels in 12 subjects. Upgaze phasically and degree-dependently increased deoxyhemoglobin level at Fp1 and Fp2, whereas downgaze phasically decreased it in 16 subjects. Unilateral anesthetization of mechanoreceptors in the supratarsal Müller muscle used to significantly reduce trigeminal proprioceptive evocation ipsilaterally impaired the increased deoxyhemoglobin level by 60° upgaze at Fp1 or Fp2 in 6 subjects. We concluded that upgaze with strong trigeminal proprioceptive evocation was sufficient to phasically activate sympathetically innervated sweat glands and appeared to induce rapid oxygen consumption in the ventromedial prefrontal cortex and to rapidly produce deoxyhemoglobin to regulate physiological arousal. Thus, eyelid opening with trigeminal proprioceptive evocation may activate the ventromedial prefrontal cortex via the mesencephalic trigeminal nucleus and locus coeruleus.

## Introduction

**Fig 1:**
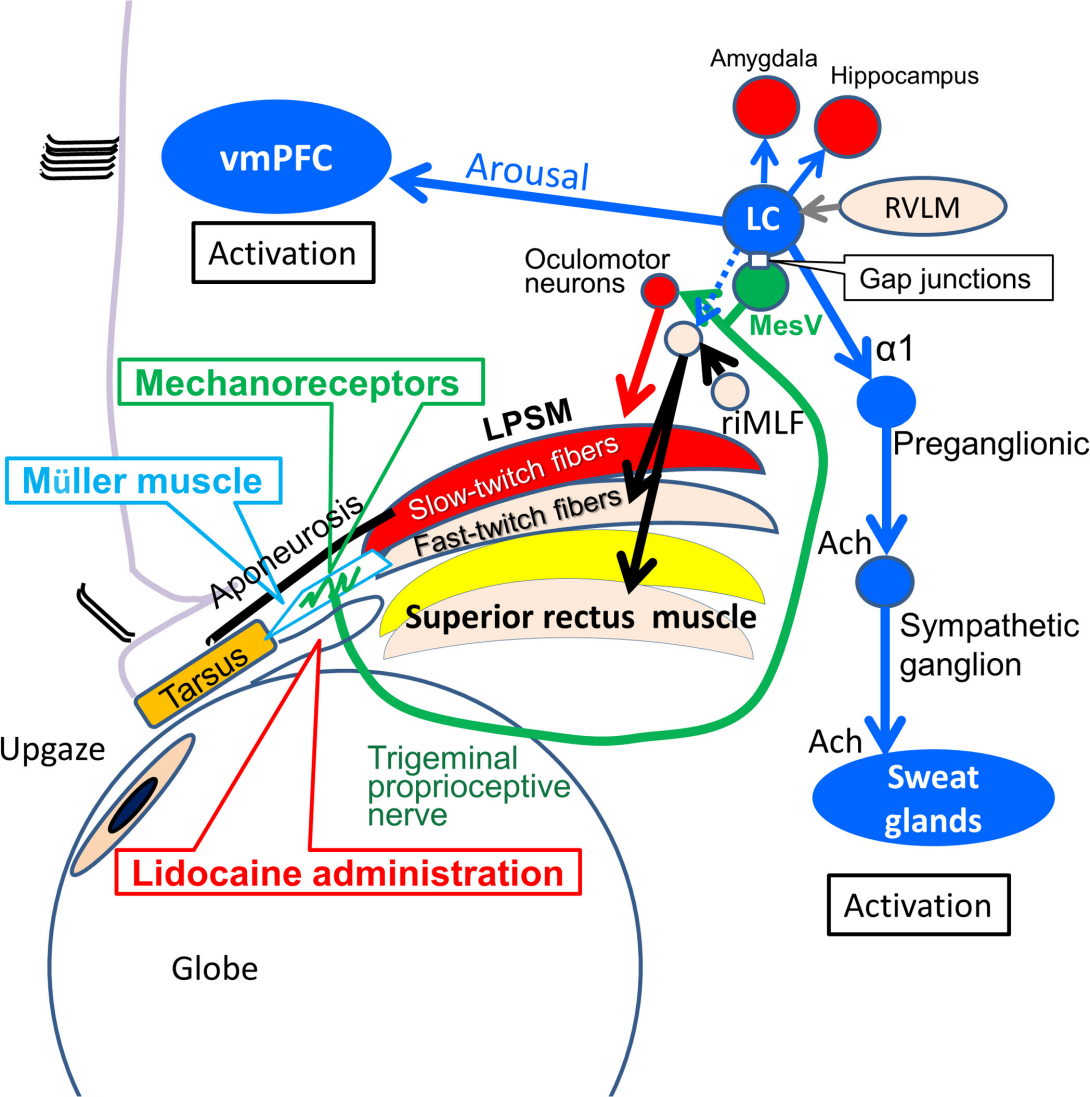
Neuroanatomy of upgaze with trigeminal proprioceptive activation to regulate a brainstem arousal mechanism. Black arrows indicate voluntary contractions of the superior rectus and levator palpebrae superioris (LPSM) fast-twitch fibers via the rostral interstitial nucleus of the medial longitudinal fasciculus (riMLF) and the oculomotor neurons. A red arrow indicates involuntary reflex contractions of the LPSM slow-twitch fibers. Green arrows indicate trigeminal proprioceptive stimulations to induce reflex contractions of the LPSM slow-twitch muscle fibers and stimulate the locus coeruleus (LC) via the mesencephalic trigeminal nucleus (MesV) through gap junctions. Blue arrows indicate efferent stimulations from the locus coeruleus (LC) to activate the ventromedial prefrontal cortex (vmPFC), palmar sweat glands, amygdala, and hippocampus. A blue dotted arrow indicates stimulation from the LC to the oculomotor neurons. The gray arrow indicates an afferent stimulation from the rostral ventrolateral medulla (RVLM) to the LC.

We previously reported temporary or permanent blepharoptosis and brow ptosis after Müller muscle resection, lacrimal gland tumor resection with the lacrimal nerve, anesthesia of the Müller muscle or branches of the lacrimal nerve, and blowout fracture due to external force to the eyelid and globe [1–4]. We also previously reported that intraoperative stretching of the Müller muscle electromyographically induces an involuntary reflex contraction of the ipsilateral levator palpebrae superioris muscle (LPSM) [5]. This contraction appeared to correspond with the periodontal jaw muscle reflex as a model of reflex contractions of the LPSM slow-twitch fibers based on extrinsic mechanoreceptor stretching. Voluntary contractions of the jaw-closing fast-twitch muscle fibers stretch the extrinsic periodontal mechanoreceptor to evoke trigeminal proprioception, which in turn continuously induces reflex contractions of the jaw-closing slow-twitch muscle fibers via the mesencephalic trigeminal nucleus [6–9]. Thus, we have been exploring the extrinsic mechanoreceptors in the Müller muscle that induce reflex contractions of slow-twitch fibers in the LPSM and frontalis muscle to tonically sustain eyelid and eyebrow positions against gravity as well as elastic resistance [10] and its related mesencephalic trigeminal proprioceptive nucleus. The supratarsal Müller muscle, which is located between the LPSM and tarsus under the levator aponeurosis (Fig 1), is innervated not only by sparse, unmyelinated sympathetic efferent fibers but also by abundant, myelinated trigeminal proprioceptive afferent fibers [1, 2, 11]. The trigeminal proprioceptive fibers innervate the intramuscular connective tissues, including the interstitial cells of Cajal [11], which are like a palisade among the smooth muscle fibers and converge as a transverse nerve that runs on the proximal Müller muscle to join the lacrimal branch of the ophthalmic trigeminal nerve in humans [1]. Because the trigeminal proprioceptive neurons are connected to each other with gap junctions [12], Fluorogold, which passes through gap junctions, was applied as a tracer to the proximal stump of the trigeminal proprioceptive nerve in rats [3]. Fluorogold densely labeled the cell bodies of the trigeminal proprioceptive neurons in the ipsilateral mesencephalic trigeminal nucleus and a part of the locus coeruleus but not in any other rat brain region, including the trigeminal ganglion.

We have confirmed the presence of a Hoffmann reflex in the LPSM and frontalis slow-twitch muscle fibers [6]. In humans, unilateral electrical stimulation to the trigeminal proprioceptive fibers innervating the mechanoreceptors in the supratarsal Müller muscle induces the following: a phasic short-latency response in the ipsilateral LPSM via the mesencephalic trigeminal nucleus and oculomotor neurons (Fig 1) [13, 14], a phasic short-latency response in the ipsilateral frontalis muscle via the mesencephalic trigeminal nucleus and facial motor neurons, and tonic long-latency bilateral responses in frontalis muscles with ipsilateral dominance via the mesencephalic trigeminal nucleus, an unknown nucleus at the reticular formation, and facial motor neurons [15]. Regarding the clinical significance of this finding, we previously proposed [5, 16–21] that during primary gaze, weak proprioceptive evocation via mild stretching of the mechanoreceptors in Müller's muscle invoked by voluntary contractions of the LPSM and superior rectus fast-twitch fibers induced reflex contractions of the LPSM slow-twitch fibers to sustain eyelid position against gravity and elastic resistance [10, 22, 23] (Fig 1). We further proposed that during upgaze (Fig 1), increased proprioceptive evocation by increased stretching of these mechanoreceptors would enhance reflex contractions of the LPSM slow-twitch fibers and evoke reflex contractions of the frontalis slow-twitch fibers to sustain eyelid and eyebrow positions against gravity and elastic resistance [10].

The cyclic adenosine monophosphate/protein kinase A (cAMP/PKA) pathway in the mesencephalic trigeminal neurons ensures long-term excitability [24]. Continuous evocation of trigeminal proprioception may involuntarily maintain continuous reflex contractions of the LPSM and frontalis slow-twitch fibers to sustain eyelid and eyebrow positions against gravity and elastic resistance, depending on the degree of mechanoreceptor stretching in the supratarsal Müller muscle. In humans, the mesencephalic trigeminal neurons comingle with cells in the locus coeruleus on both sides of the mesencephalic tract [25]. According to our anterograde tracing study in rats [3], the cell bodies of trigeminal proprioceptive neurons potentially make gap-junctional connections with cells of the locus coeruleus, which is implicated in arousal and autonomic function [26]. Thus, we hypothesized that eyelid opening with trigeminal proprioceptive activation by stretching mechanoreceptors in the supratarsal Müller muscle could stimulate the locus coeruleus and is an arousal reaction, as suggested by its use to diagnose coma or impaired consciousness [27].

A direct regulatory relationship between the ventromedial prefrontal cortex activity and skin conductance measurements has been reported as a useful physiological index of arousal [28, 29]. To clarify functional connectivity among the mesencephalic trigeminal nucleus, locus coeruleus, and ventromedial prefrontal cortex in arousal and autonomic function, we assessed whether upgaze with enhanced evocation of the trigeminal proprioception via mechanoreceptor stretching activates sympathetically innervated sweat glands and the ventromedial prefrontal cortex (Fig 1).

## Materials and Methods

### Subjects

Sixteen healthy subjects (5 female and 11 male plastic surgeons; 35.5 ± 5.1 years old) were enrolled in the study to assess palmar sweating responses and prefrontal cortex hemodynamic changes. The study protocol was approved by the Committee for Medical Ethics of Shinshu University School of Medicine. All subjects were fully informed about the study and provided written consent prior to participation.

### Palmar sweating responses induced by 60° upgaze for 5 s

To investigate whether tonic upgaze induces palmar sweating, 16 subjects were instructed to perform a 60° upgaze toward a target mark on the wall for 5 s without tilting the head backwards. Before and after the task, subjects were asked to keep a 45° downward gaze at a target mark on the wall without inclining the head.

Active palmar sweating in all subjects was measured using a perspiration meter (SKN-2000; Skinos, Nagoya, Japan) attached to the left and right palmar surfaces of the thumbs (contacted area 1 cm2) [30]. The absolute amount of palmar perspiration was recorded as loss of water per constant area and time on a chart recorder. When the 60° upgaze was initiated or completed, reference marks were made on the chart recorder. However, the integrated absolute amount during the 5-s 60° upgaze was calculated for 5 s from the point at which the active increase in water loss began, and these values were compared with the integrated absolute amounts of water loss for 5 s before the 60° upgaze (as a control).

### Depiction of the activated ventromedial prefrontal cortex by 60° upgaze for 10 s

To investigate whether tonic upgaze activates the ventromedial prefrontal cortex, 12 of 16 subjects were instructed to perform a 60° upgaze at a target mark on the wall for 10 s without tilting the head backwards. In 4 subjects who had insufficient sleep the previous night, upgaze did not induce an increase in palmar sweating. These subjects also had difficulty maintaining 60° upgaze for 10 s with heavy headgear in place. Therefore, they were excluded from the prefrontal cortex task. Trials were not performed in the same day, and the task was preceded and followed by a 45° downward gaze at a target mark on the wall.

Activation of the ventromedial prefrontal cortex by tonic upgaze was confirmed by measuring prefrontal cortex hemodynamic changes with functional near-infrared spectroscopy (fNIRS). The fNIRS system consisted of a multichannel optical topography system (FOIRE-3000, Shimadzu Co., Kyoto, Japan) [31]. Fourteen light-emission and 14 light-detection probes were arranged in a 4 × 7 rectangular lattice. Each recording channel consisted of one light-emission probe and one light-detection probe located 3 cm away from each other to measure cerebral hemodynamics. Headgear with 28 mounted probes for 45 recording channels was placed securely on the scalp overlying the prefrontal area. The light-detection probe between channels 16 and 17 was located on the Fz, and the lowest probes were positioned along the Fp1–Fp2 line according to the international 10–20 system of electroencephalogram electrode placement [32]. Each light-emission probe was connected to three laser diodes with wavelengths of 780, 805, and 830 nm.

To compare relative values of deoxyhemoglobin, oxyhemoglobin, and total hemoglobin, their values at the start of the 45° downward gaze was set as 0. Concentration changes (mM × cm) in deoxyhemoglobin, oxyhemoglobin, and total hemoglobin during the last 9 s of the 10-s 60° upgaze task were integrated to detect prefrontal cortex activation in each of 12 subjects. Because the probes could slip if the subjects shifted from the 45° downward gaze to 60° upgaze, the concentration changes during the first 1 s of the task were not included to solve the problem of headgear slippage. To depict activated areas in the ventromedial prefrontal cortex, the integrated concentration changes in deoxyhemoglobin, oxyhemoglobin, and total hemoglobin were accumulated from all of 12 subjects, respectively.

### Degree-dependent activation of the ventromedial prefrontal cortex during eyelid retraction

To investigate whether the degree of eyelid retraction is associated with degree-dependent hemodynamic activation in the ventromedial prefrontal cortex, we examined changes in the relative concentrations of deoxyhemoglobin, oxyhemoglobin, and total hemoglobin at Fp1 and Fp2 [33] with lightweight wireless transcranial near-infrared optical spectroscopy [34], according to the international 10–20 system [32].

A two-channel wireless system (Pocket NIRS Duo; Dynasense Inc., Hamamatsu, Japan) was used to measure concentration changes (mmol/L × cm) in deoxyhemoglobin and oxyhemoglobin contents every 100 ms at Fp1 and Fp2. The equipment consisted of two photodiodes as detectors and six light-emitting diodes with three wavelengths (735, 810, and 850 nm) as light sources. The distance between the detection probe and the corresponding emission probe was 3 cm. The probes were fixed with double-sided tape and a wide elastic bandage to avoid slippage during examinations. To compare concentration changes in deoxyhemoglobin, oxyhemoglobin, and total hemoglobin, their concentrations at the start of the 45° downward gaze was set at 0. Without trials in the same day to avoid prolonged changes in hemoglobin levels, the head was kept in an upright position, while the degree of eyelid retraction was changed as follows: downgaze at 45° for 60 s, 0° (primary position) for 5 s, 30° upgaze for 5 s, and 60° upgaze for 5 s during the tasks without inclining the head or tilting the head backwards. Each increment of eyelid retraction from the primary position to 60° upgaze was preceded by a 45° downgaze for more than 30 s to ensure constant visual input. Subjects gazed at target figures marked on a gray wall in front of them corresponding with 45° downgaze, 0° (primary position), 30°, and 60° upgaze. When the degree of eyelid retraction was changed, reference marks were recorded on the tracings. The examination began with the subjects sitting in a comfortable position in front of the gray wall to keep visual stimulation constant. Although relative values of deoxyhemoglobin, oxyhemoglobin, and total hemoglobin were measured chronologically in the tracing, only the integrated relative values of deoxyhemoglobin for the various degrees of eyelid retraction, which indicate direct activation of the ventromedial prefrontal cortex, were subjected to statistical comparisons. Deoxyhemoglobin values were integrated for 5 s during the primary gaze (0°), 30° upgaze, and 60° upgaze, but those during the 45° downgaze for 5 s prior to the primary gaze (0°) were adopted among the four 45° downgazes. The deoxyhemoglobin values during changes in eyelid retraction were compared.

### Impaired activation of the ipsilateral ventromedial prefrontal cortex by unilateral anesthetization of mechanoreceptors in the supratarsal Müller muscle

To investigate whether the act of upgaze causes an increase in arousal due to the proprioceptive feedback, six subjects were instructed to perform the same task as performed in the degree-dependent activation of the ventromedial prefrontal cortex during eyelid retraction before and after unilateral anesthetization of mechanoreceptors in the supratarsal Müller muscle. According to the results before the anesthetization, the eyelid on the side of more activated ventromedial prefrontal cortex was anesthetized. Subjects, who wore a 2-channel NIRS on their forehead, laid in a supine position, raised their chin, and gazed downward. The upper eyelid was detached from the globe by an eyelid retractor for at least 75 seconds to create a space in the upper fornix. Ten to 12 drops of 4% lidocaine were administered into the space, and the lidocaine was retained in this position by gravity to exclusively anesthetize the unilateral supratarsal Müller smooth muscle fibers through the conjunctiva palpebrae (Fig 1). Before and after lidocaine administration in six subjects, we examined changes in the relative concentrations of deoxyhemoglobin, oxyhemoglobin, and total hemoglobin at Fp1 and Fp2 with a two-channel NIRS. Specifically, differences in the integrated deoxyhemoglobin values between 60° upgaze for 5 s and 45° downgaze for 5 s prior to the primary gaze (0°) were compared before and after anesthetization.

### Statistical analysis

Data were analyzed using Wilcoxon signed rank tests, one-way analyses of variance (ANOVAs) with Bonferroni multiple comparison procedures, and paired t-tests. All tests were conducted with SPSS statistics 22 (IBM Corporation, Armonk, NY). A *P* value less than 0.05 was considered to indicate a significant difference. All data are presented as the mean ± SD.

## Results

### Palmar sweating response by 60° upgaze for 5 s

**Fig 2:**
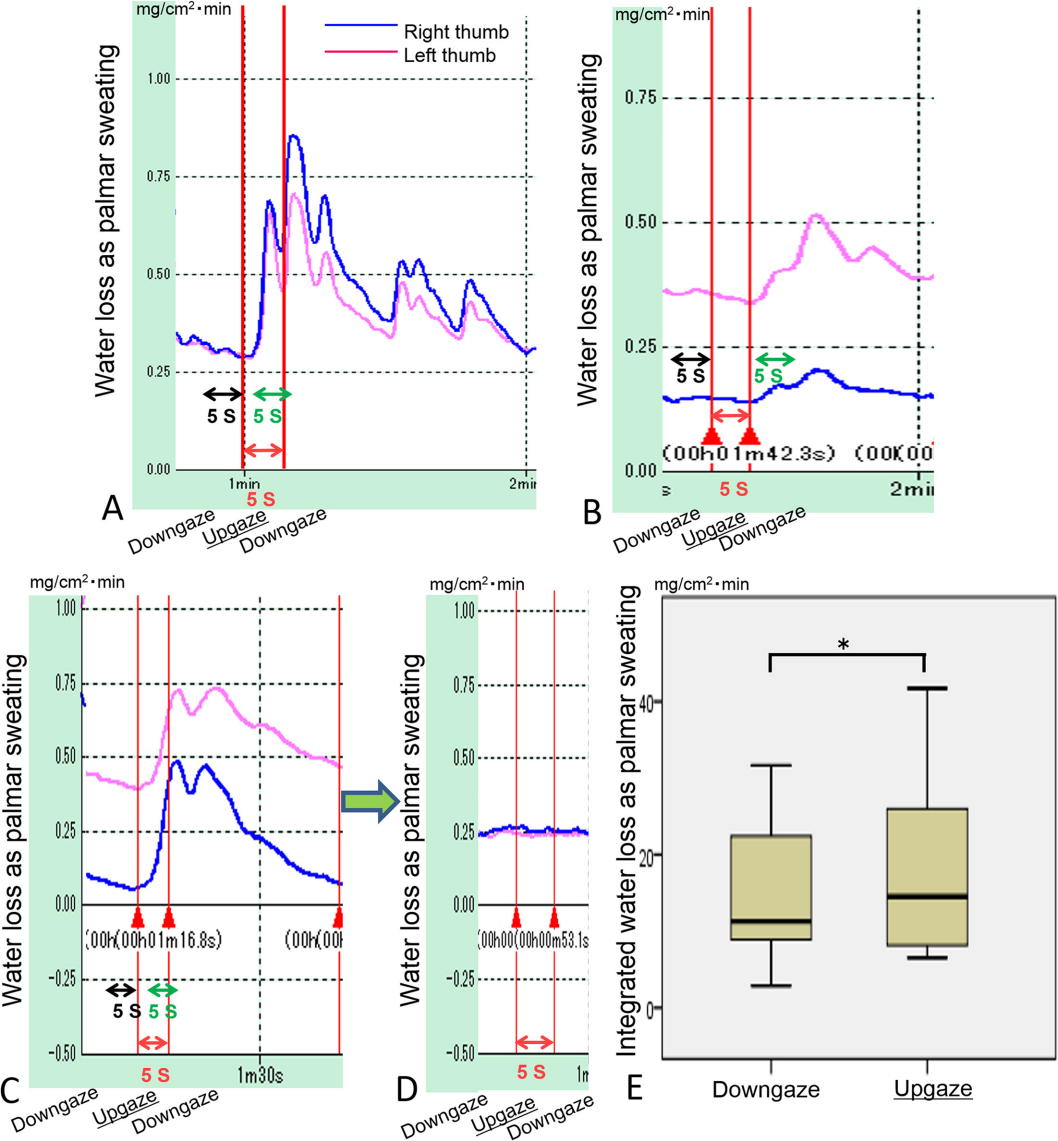
Palmar sweating response during a 60° upgaze for 5 s. (**A**–**C**) Three representative results showing various palmar sweating patterns. (**D**) No palmar sweating response in the same subject (**C**) on another day when he had insufficient sleep time. (**E**) The integrated absolute amounts of water loss during 5-s of 60° upgazes were compared with those in the preceding 5-s of 45° downgaze (*P < 0.05).

Palmar sweating responses commenced 1 to 6 s after initiating a 60° upgaze and increased rapidly without a plateau in 12 of 16 subjects (Fig 2A–2C). It decreased 1 to 9 s after beginning downgaze and persisted over a period of approximately 40 to 110 s. Increases in palmar sweating responses were not measured in 4 of 16 subjects who had insufficient sleep time. In the subjects with positive palmar sweating responses, 60° upgaze for 5 s did not induce sweating responses on another day when they had insufficient sleep time (Fig 2D). The median integrated absolute amount of water loss during 60° upgaze for 5 s (12.92 mg/cm2/min) was significantly larger (*P* < 0.05) than that for 5 s before 60° upgaze (10.79 mg/cm2/min) (Fig 2E).

### Depiction of the activated ventromedial prefrontal cortex by 60° upgaze for 10 s

**Fig 3:**
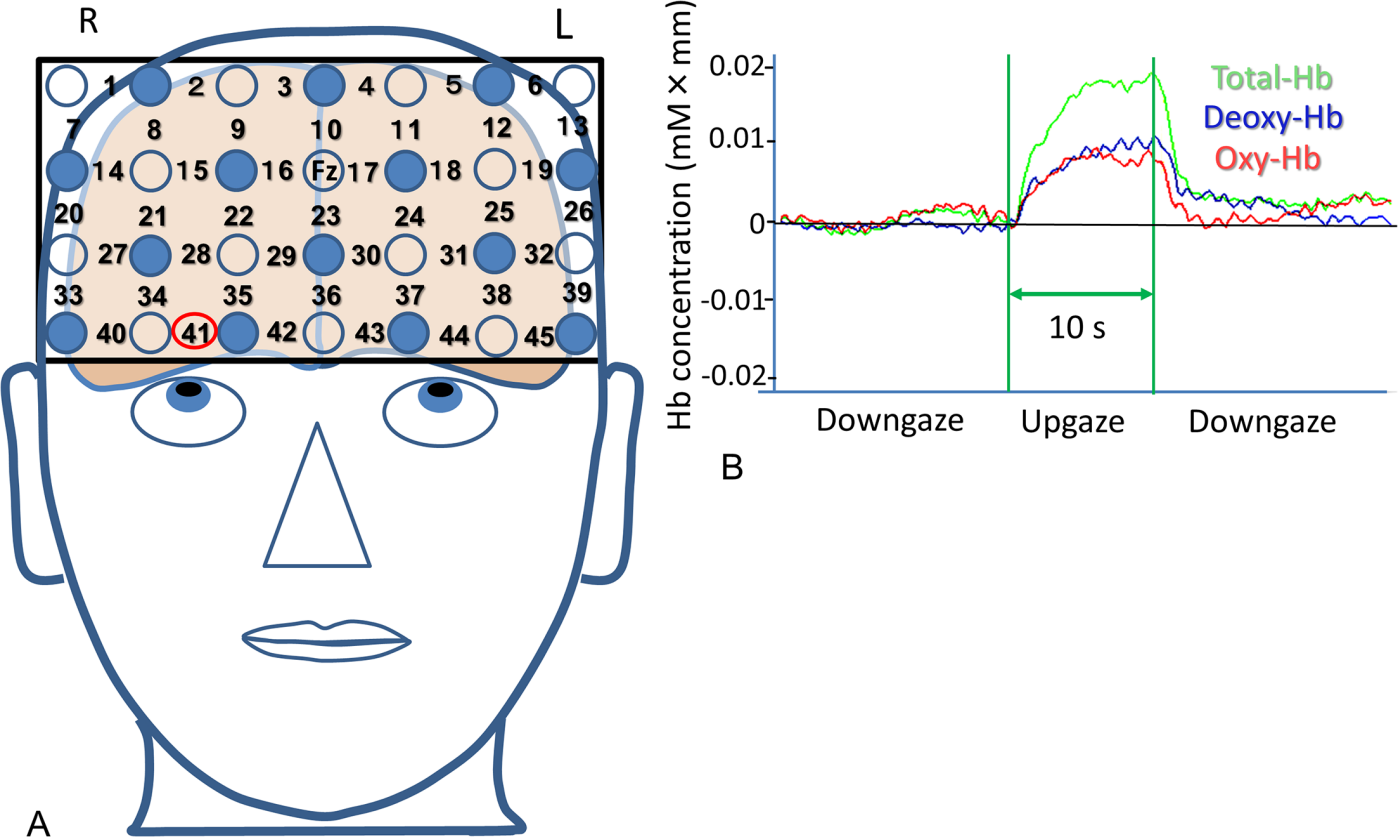
Measurement of hemodynamic changes in the prefrontal cortex with functional near-infrared spectroscopy. (**A**) Headgear with 28 mounted probes for 45 recording channels is securely placed on the scalp overlying mainly the prefrontal area while participants performed the 60° upgaze. The light-detection probe between channels 16 and 17 was located on the Fz, and the lowest probes were positioned along the Fp1–Fp2 line. (**B**) Typical concentration changes in deoxyhemoglobin (Deoxy-Hb), oxyhemoglobin (Oxy-Hb), and total hemoglobin (Total-Hb) during the 10-s 60° upgaze task at channel 41.

**Fig 4:**
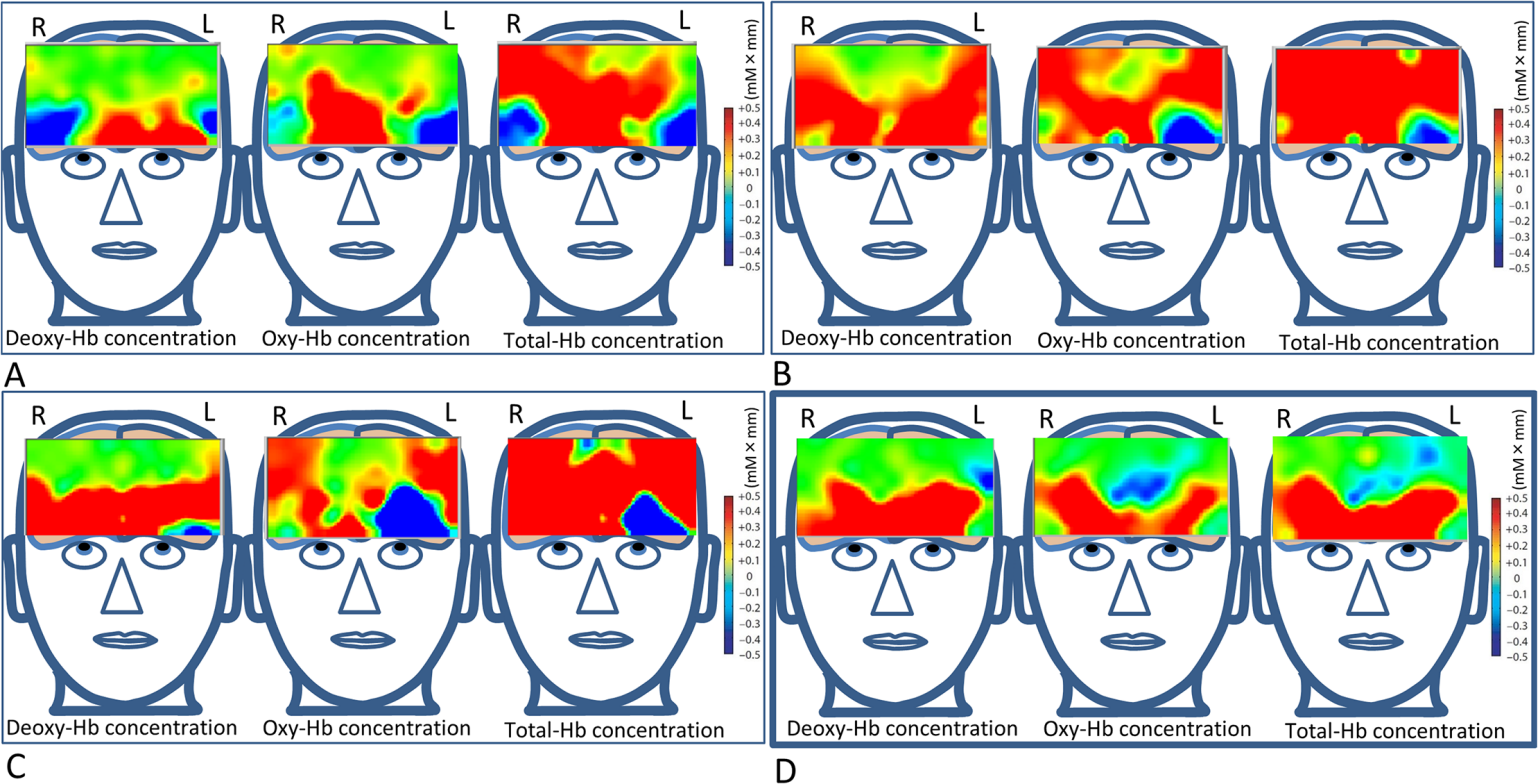
Depiction of the activated ventromedial prefrontal cortex during a 10-s 60° upgaze as measured by functional near-infrared spectroscopy. (**A**–**C**) Three representative examples show various activated prefrontal cortex areas due to the integrated concentration changes in deoxyhemoglobin (Deoxy-Hb), oxyhemoglobin (Oxy-Hb), and total hemoglobin (Total-Hb). (**D**) The accumulation of the integrated concentration changes from all 12 subjects depicts the ventromedial prefrontal cortex activation in deoxyhemoglobin (Deoxy-Hb), oxyhemoglobin (Oxy-Hb), and total hemoglobin (Total-Hb) concentrations.

At many recording channels (Fig 3A), deoxyhemoglobin, oxyhemoglobin, and total hemoglobin levels were phasically increased by a 60° upgaze for 10 s, and they were phasically decreased when subjects performed a 45° downgaze (Fig 3B). The integrated concentration changes in deoxyhemoglobin, oxyhemoglobin, and total hemoglobin during 60° upgaze for the last 9 s showed a variety of prefrontal cortex activations in each of the subjects (Fig 4A–4C). The accumulation of the integrated concentration changes from all 12 subjects appeared to depict overall activation of the ventromedial prefrontal cortex in each of the deoxyhemoglobin, oxyhemoglobin, and total hemoglobin concentrations (Fig 4D).

### Degree-dependent ventromedial prefrontal cortex activation by changes in eyelid retraction

**Fig 5:**
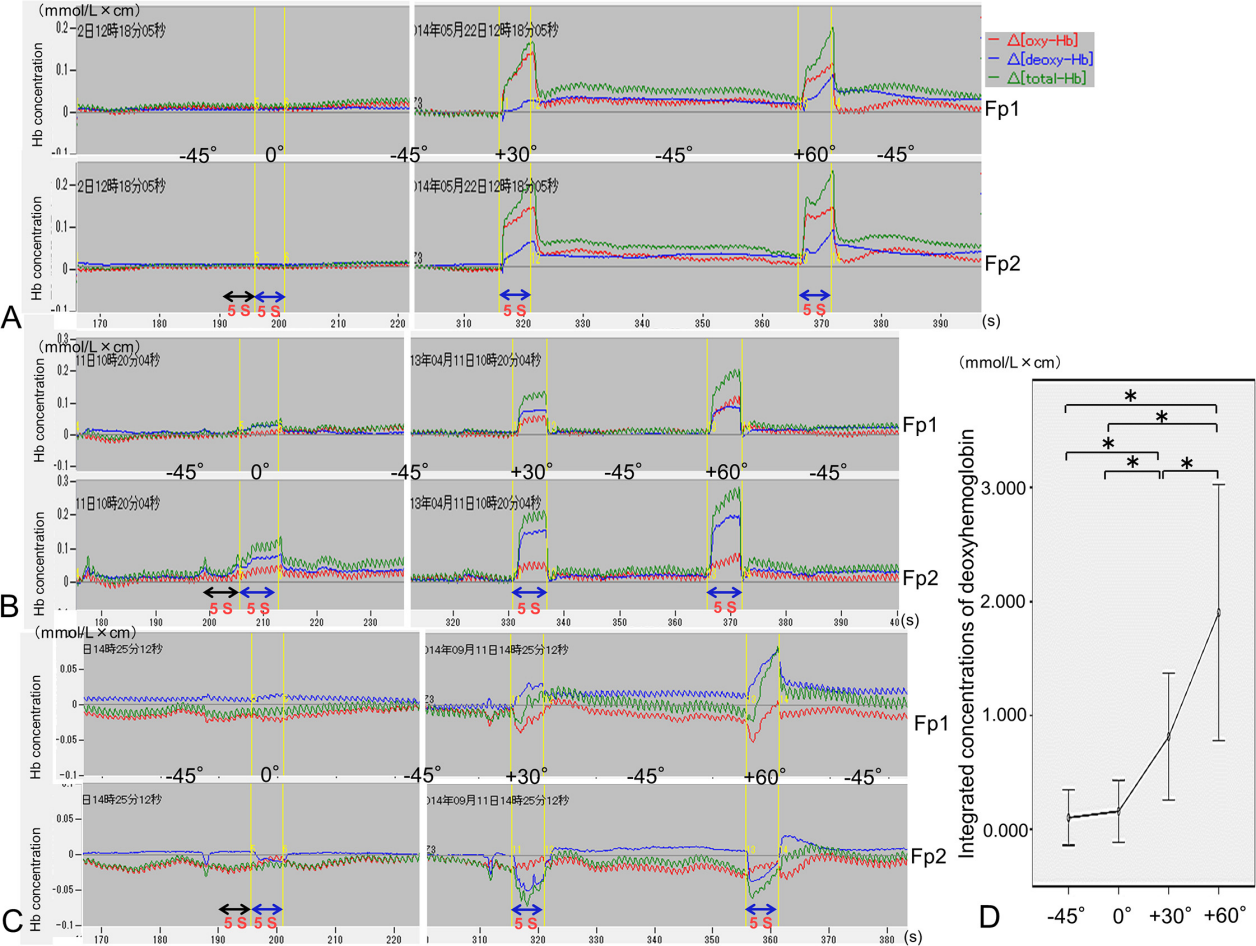
Degree-dependent ventromedial prefrontal cortex activation following changes in the degree of eyelid retraction. Relative changes in the deoxyhemoglobin, oxyhemoglobin, total hemoglobin concentrations (mmol/L × cm) were measured at Fp1 and Fp2 according to the changes in the degree of eyelid retraction such as 45° downgaze (-45°), primary gaze (0°), 30° upgaze (+30°), and 60° upgaze (+60°). Three representative examples show bilateral activation for 5-s 30° upgaze (+30°) (**A**), 5-s asymmetrical bilateral activation from primary gaze (0°) (**B**), and unilateral activation and contralateral deactivation (**C**). (**D**) The integrated concentration of deoxyhemoglobin for 5 s was significantly and degree-dependently increased from the 30° upgaze (*P < 0.05).

There were three patterns of deoxyhemoglobin, oxyhemoglobin, and total hemoglobin concentration changes that corresponded to the degree of eyelid retraction in 16 subjects. Deoxyhemoglobin, oxyhemoglobin, and total hemoglobin concentrations were degree-dependently and symmetrically increased at both Fp1 and Fp2 sites in 12 subjects (Fig 5A) and asymmetrically in 1 subject (Fig 5B). Although there were degree-dependent increased in these parameters at the unilateral site, they were degree-dependently decreased at the contralateral site in 3 subjects (Fig 5C). Downgazing after 30°or 60° upgazing elicited phasic decreases in deoxyhemoglobin concentrations. Nevertheless, tonic degree-dependent increases in deoxyhemoglobin concentrations were sustained compared to those during downgaze before 30° or 60° upgazing (Fig 5A–5C).

The changes in deoxyhemoglobin concentration at the dominant or positive sites were analyzed. The integrated deoxyhemoglobin concentrations during 45° downgaze (0.103 ± 0.243 mmol/L × cm), primary gazing (0.158 ± 0.271 mmol/L × cm), 30° upgazing (0.814 ± 0.556 mmol/L × cm), and 60° upgazing (1.903 ± 1.124 mmol/L × cm) were significantly different (F[1.323, 19.844] = 37.315, MSe = 0.681, *P* < 0.01: adjusted by Greenhouse-Geisser correction).

The integrated deoxyhemoglobin concentration during primary gaze was not significantly larger than that of the 45° downgaze. However, all the other deoxyhemoglobin values were significantly different from each other in a degree-dependent manner (*P* < 0.05, Fig 5D).

### Impaired activation of the ipsilateral ventromedial prefrontal cortex by unilateral anesthetization of mechanoreceptors in the supratarsal Müller muscle

**Fig 6:**
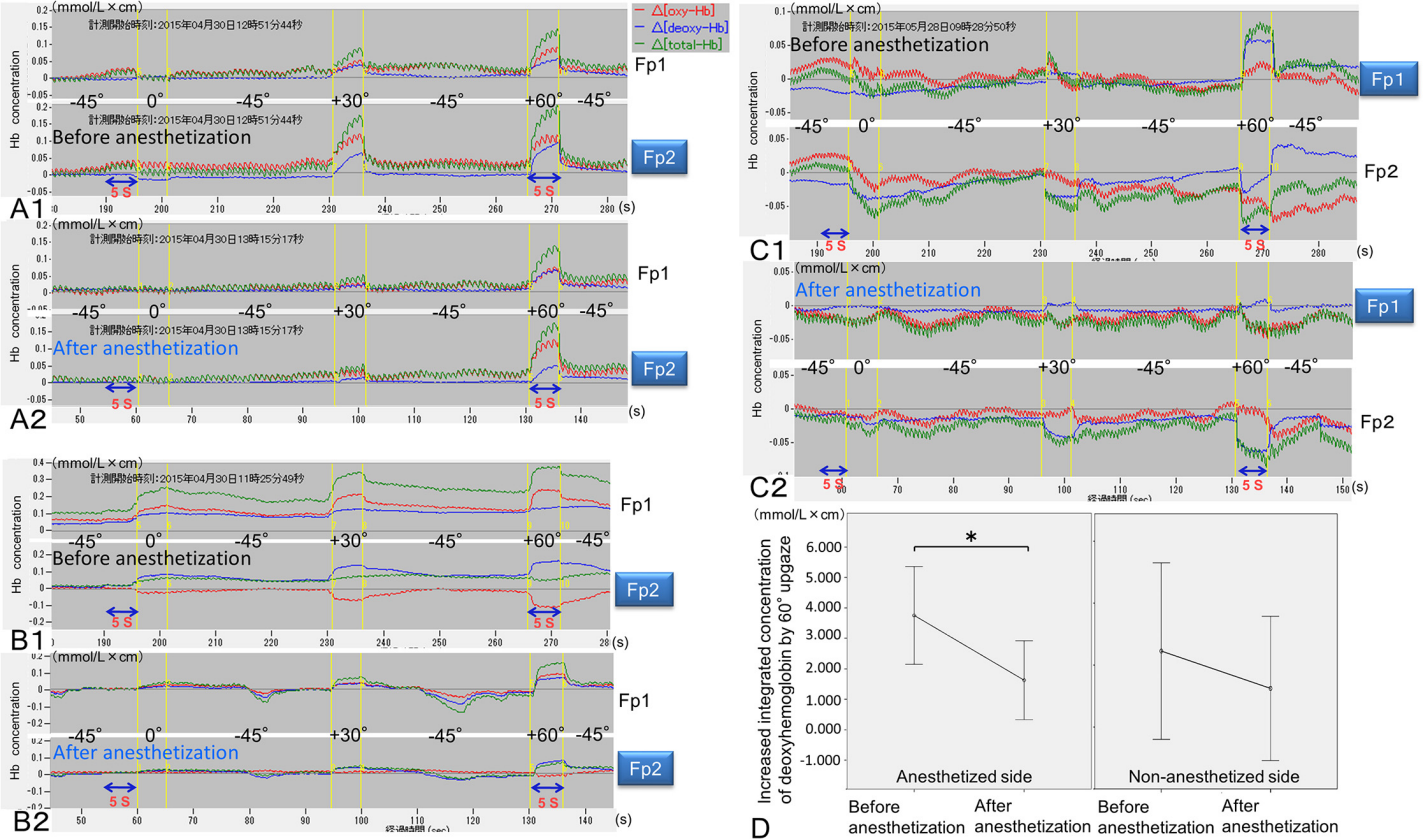
Impaired activation of the ipsilateral ventromedial prefrontal cortex by unilateral anesthetization of mechanoreceptors in the supratarsal Müller muscle. Relative changes in the deoxyhemoglobin, oxyhemoglobin, total hemoglobin concentrations (mmol/L × cm) were measured at Fp1 and Fp2 according to the changes in the degree of eyelid retraction such as 45° downgaze (-45°), primary gaze (0°), 30° upgaze (+30°), and 60° upgaze (+60°) before (**A1, B1,** and **C1**) and after (**A2, B2,** and **C2**) anesthetization. Before anesthetization, three representative examples show bilateral activation (**A1**), asymmetrical bilateral activation (**B1**), and unilateral activation and contralateral deactivation for 5 s of 60° upgaze (+60°) (**C1**). After anesthetization, all of the examples show impaired activation at Fp1 or Fp2 on the anesthetized side (**A2**, **B2**, and **C2**). (**D**) On the anesthetized side, the increased integrated concentration of deoxyhemoglobin by 60° upgaze for 5 s after anesthetization was significantly smaller than that before anesthetization (*P < 0.01). However, on the non-anesthetized side, the increased integrated concentration of deoxyhemoglobin by 60° upgaze for 5 s after anesthetization was not significantly smaller than that before anesthetization.

On the anesthetized side of more activated ventromedial prefrontal cortex by upgazes, the mean increase in the integrated deoxyhemoglobin values between 60° upgaze for 5 s and 45° downgaze for 5 s prior to the primary gaze (0°) after ipsilateral anesthetization (1.626 ± 1.298 mmol/L × cm) was significantly smaller than that before anesthetization (3.7554 ± 1.606 mmol/L × cm) (*P*<0.01) (Fig 6D). Conversely, on the non-administered side of less activated ventromedial prefrontal cortex by upgazes, although one subject showed degree-dependent decreases in deoxyhemoglobin concentrations by upgazes (Fig 6C1 and 6C2), the mean increase in the integrated deoxyhemoglobin values between 60° upgaze for 5 s and 45° downgaze for 5 s prior to the primary gaze (0°) after contralateral anesthetization (2.454 ± 2.852) was not significantly smaller than that before contralateral anesthetization (1.248 ± 2.328) (Fig 6D). Before anesthetization, tonic degree-dependent increases in deoxyhemoglobin concentrations were sustained compared to those during downgaze before 30°or 60° upgazing (Fig 6B1 and 6C1). However, after anesthetization, they were not sustained (Fig 6B2 and 6C2).

## Discussion

Upgaze strongly evokes trigeminal proprioception and phasically activates sympathetically innervated sweat glands with a short delay. Conversely, downgaze halts trigeminal proprioceptive activity [35] and gradually produces decreased sweating after a delay. Because the mesencephalic trigeminal neurons [12] and locus coeruleus neurons are reciprocally connected [36], phasic sweating after upgaze may indicate synchronous activation of the mesencephalic trigeminal and locus coeruleus neurons via connecting gap junctions. Because the locus coeruleus sends direct projections to not only the amygdala and preganglionic sympathetic neurons in the intermediolateral cell column of the spinal cord [37–40] but also the ventromedial prefrontal cortex reported here, the amygdala and ventromedial prefrontal cortex could be simultaneously involved in regulating physiological arousal with sympathetic, cholinergic sweating (Fig 1) [41–44]. Because the preganglionic sympathetic neurons send sympathetic and cholinergic inputs to sweat glands via postganglionic sympathetic neurons, the neural routes from the trigeminal proprioceptive neurons to the palmar sweat glands are polysynaptic. Thus, the delays between trigeminal proprioceptive evocation and palmar sweating responses can vary. Changes from positive to negative palmar sweating patterns in the same subjects between the days when they had sufficient sleep the previous night and the days when they had insufficient sleep might be caused by impaired mechanoreceptor stretching to strongly evoke proprioception during the 5-s 60° upgaze task and/or a norepinephrine deficit.

Using a dipole tracing method, memory retrieval or mental calculation was found to activate the prefrontal cortex, amygdala, and hippocampus with induction of palmar sweating. It also induces large waves on electroencephalograms prior to palmar sweating. The origin of the large waves was estimated to be around the orbits, reflecting activity of the eyelid or eye muscles [45–47]. These reports suggest that the sweating response induced by mental activity is preceded by contractions of the eyelid or eye muscles. These may stretch the mechanoreceptors in the supratarsal Müller muscle to evoke trigeminal proprioception that stimulates the locus coeruleus, which in turn activates the ventromedial prefrontal cortex, amygdala, hippocampus, and sympathetically innervated sweat glands during memory retrieval or mental calculation (Fig 1).

According to theoretical modeling and experimental data regarding blood flow and oxygen delivery to the human brain during functional activity, an increase in oxygen consumption due to neural activation occurs primarily in the brain tissue and secondarily in the blood because an intact brain tissue has excess oxygen delivery compared to utilization [48]. The 60° upgaze with strong evocation of trigeminal proprioception, which was enough to phasically activate sympathetically innervated sweat glands, appeared to also rapidly consume oxygen in the brain tissue and blood, producing deoxyhemoglobin. The rapid increases in oxyhemoglobin and total hemoglobin during 60° upgaze appeared to be due to a large rapid influx of oxyhemoglobin and total hemoglobin in relation to the magnitude of the deoxyhemoglobin increase. Because oxyhemoglobin and total hemoglobin appeared to be secondarily increased by vasodilatation mediated by local mechanisms [49, 50], we focused on deoxyhemoglobin concentration changes in this study.

The fNIRS also may have measured the combined activity of the frontalis muscles and the prefrontal cortex. Upgaze contracts the bilateral frontalis muscles [17–19, 21], which induce a rapid increase in deoxyhemoglobin, rapid decrease in oxyhemoglobin, and slow increase in total hemoglobin [51]. The patterns in the fNIRS recordings did not match the distribution of the frontalis muscles with contraction [17]. Additionally, three subjects exhibited unilateral decreases in deoxyhemoglobin concentration at the Fp1 or Fp2 on the frontalis muscles with contraction. However, the frontalis muscle vascular changes must be considered if oxygen consumption in the prefrontal cortex is evaluated [31].

A gaze shift of less than 15° is usually unaccompanied by a head movement in healthy subjects. The oculomotor range is approximately ± 50°, meaning that the gaze shifts beyond this degree always necessitate a head movement to reach the visual target [52]. Most likely, the 60° upgaze without tilting the head backwards made sense in terms of strong, trigeminal proprioceptive activation that could visualize the phasic induction of palmar sweating and the hemodynamic changes in the prefrontal cortex.

According to the integrated deoxyhemoglobin, oxyhemoglobin, and total hemoglobin concentration changes from the fNIRS recordings during 60° upgaze for 10 s, the accumulation of the integrated concentration changes from all 12 subjects appeared to depict overall activation of the ventromedial prefrontal cortex. According to changes in the degree of eyelid retraction, 45° downgazing or primary gazing did not significantly increase the integrated deoxyhemoglobin concentration, so trigeminal proprioceptive activation did not appear to consume oxygen in the blood. However, trigeminal proprioceptive activation by 30° or 60° upgazing appeared to be sufficiently strong to promote oxygen consumption with rapid deoxyhemoglobin production. The phasic increase or decrease in deoxyhemoglobin concentration at Fp1 and Fp2 elicited by 30° and 60° upgazing or subsequent downgazing indicates synchronous activation or deactivation among the mesencephalic trigeminal neurons, locus coeruleus, and ventromedial prefrontal cortex via stretching or relaxing mechanoreceptors in the supratarsal Müller muscle (Fig 1).

Regardless of whether upgaze is causing an increase in arousal due to the proprioceptive feedback or maintenance of upgaze is uncomfortable or requires increased concentration, significantly impaired activation of the ipsilateral ventromedial prefrontal cortex by unilateral anesthetization of the proprioceptive neural arc confirmed the role of the afferent activity of the proprioception (Fig 1). Because the mesencephalic trigeminal nucleus connects to the ipsilateral locus coeruleus [3] and the locus coeruleus predominantly projects onto the ipsilateral prefrontal cortex [53–56], the afferent trigeminal proprioceptive neurons appear to predominantly activate the ipsilateral ventromedial prefrontal cortex through the ipsilateral locus coeruleus.

Mammalian brains reportedly generate internal activity independent of environmental stimuli [57]. Because mammals have LPSMs [23] which evoke trigeminal proprioception by stretching mechanoreceptors in the supratarsal Müller muscle, they can use internal stimuli such as imperceptible trigeminal proprioception, which is evoked by eye and eyelid movements except those during non-rapid eye movement sleep and does not relay through the thalamus. Our results suggest that strong evocation of trigeminal proprioception by 30° and 60° upgazing appeared to elicit sustained activation of the ventromedial prefrontal cortex. Unilateral electrical stimulation of the rat locus coeruleus elicits sustained activation of the medial prefrontal cortex [58]. The modulation of the medial prefrontal cortex excitability state could be due to a norepinephrine release directly in the medial prefrontal cortex [57] or thalamus [59], or it could be mediated by activation of cholinergic ascending pathways that provide direct electrical stimulation to the locus coeruleus [60, 61]. However, the synchronous modulation of hemodynamic changes in the ventromedial prefrontal cortex elicited by upgazing or downgazing suggests direct functional connectivity among the mesencephalic trigeminal nucleus, locus coeruleus, and ventromedial prefrontal cortex. Although the locus coeruleus sends projections to all cortical regions, our results indicated that the ventromedial prefrontal cortex is activated by a portion of the locus coeruleus [62], which is connected with a part of the mesencephalic trigeminal nucleus that receives proprioceptive input from mechanoreceptors in the supratarsal Müller muscle [3].

A relationship between trigeminal sensory input and wakefulness has also been reported [63, 64]. The bilateral destruction of the trigeminal nerves [64] in both acute and chronic preparations from encéphale isolé cats, whose brainstems were severed between the medulla and spinal cord [63], led to impaired eyelid retraction and wakefulness. Sensory axons that relay discriminative touch, pain, temperature, and proprioception from the body enter the spinal cord via the dorsal root ganglia, which contain the cell bodies. However, sensory axons from the face enter the brain stem via the trigeminal ganglion that contains the cell bodies of all sensory axons except those responsible for proprioception from the mechanoreceptors in the supratarsal Müller muscle. Thus, the cell bodies of trigeminal proprioceptive neurons could be considered centrally displaced ganglion cells that transmit afferent signals from mechanoreceptors in the supratarsal Müller muscle to the mesencephalon. The central processes of these cells may be able to form excitatory synaptic connections with both oculomotor neurons and facial motor neurons and form gap-junctional connections with the locus coeruleus [3]. Because the 30° and 60° upgazes stretch the upper eyelid skin and eyelashes, the trigeminal sensory afferent from the eyelids and eyelashes that possibly elicits the blink reflex reaches the principal and spinal trigeminal nuclei. This proprioceptive or nociceptive trigeminal sensory input may also relate to wakefulness [65, 66].

Eyelid opening in patients with coma or impaired consciousness is considered an arousal reaction [27]. The presence of eyelid opening, either spontaneously or in response to verbal command or pain, indicates that the brainstem’s arousal mechanisms are active. Verbal command or pain stimulates the locus coeruleus via the rostral ventrolateral medulla [67]. A small number of cells in the locus coeruleus project to the oculomotor nuclear complex [68], and high levels of α1-adrenoceptors in this area [69–71] indicate that there is excitatory noradrenergic input to these neurons. Therefore, the slightly activated locus coeruleus may open the eyelid to increase visual input and trigeminal proprioceptive activation to enhance locus coeruleus stimulation for arousal. Analyses of eyelid and body movements using a home-based portable sleep monitoring system revealed that spontaneous eyelid movements in sleeping subjects are related to arousal, and decreased eyelid movements in awake subjects are related to decreased vigilance and sleep onset [72–74]. Thus, eyelid opening has been empirically related to arousal. Our results might also explain the oculogyric crisis characterized by tonic upward deviation of the eyes owing to a side effect of neuroleptic drug treatment [75] or absence seizures in children [76, 77]. Tonic upgaze in these patients may activate the ventromedial prefrontal cortex to enhance the physiological arousal.

The relationship between visual attention and consciousness has been investigated to understand the mechanisms and purpose of visual attention and to uncover the neural basis of consciousness and the subjective mind [78, 79]. The neuronal correlates of (visual) consciousness constitute the minimal set of neuronal events and mechanisms that are jointly sufficient for a specific conscious visual perception, and these mechanisms involve the firing of forebrain neurons. Visual attention apparently consists of both visual input to the primary visual cortex and fixation. The mesencephalic trigeminal neurons that provide input to locus coeruleus cells will be activated when the eyelids are opened for visual fixation. Thus, increased lid mechanoreceptor stretching may parallel one of the neuronal correlates of visual consciousness. Given the rapid activation of the locus coeruleus in response to biologically significant events that evoke simple behavioral and autonomic reflexes, it is likely these phenomena are driven by a common input [80]. In the simplest possible scenario (Fig 1), the mesencephalic trigeminal nucleus controls the activation of the locus coeruleus with autonomic arousal if triggered by arousing stimuli [81, 82], such as the trigeminal proprioception that is commonly evoked by eyelid opening and enhanced by upgaze.

When the levator aponeurosis is disinserted from the tarsus in blepharoptosis [16], increased voluntary contractions of fast-twitch fibers in the LPSM and superior rectus muscle [83, 84] increasingly stretch mechanoreceptors in the supratarsal Müller muscle. This process results in tonic reflex contractions of the occipitofrontalis slow-twitch muscle fibers via the mesencephalic trigeminal nucleus connection to facial motor neurons to sustain eyebrow positions against gravity. These contractions lead to chronic tension-type headaches [15, 17, 18, 21]. Primary gaze in patients with aponeurosis-disinserted blepharoptosis corresponds to 30°or 60° upgaze in subjects without aponeurosis-disinserted blepharoptosis in terms of trigeminal proprioceptive activation by stretching mechanoreceptors in the supratarsal Müller muscle (Fig 1). Thus, the locus coeruleus and ventromedial prefrontal cortex in patients with aponeurosis-disinserted blepharoptosis are stimulated by the mesencephalic trigeminal nucleus during eyelid retraction to a greater degree than in subjects without aponeurosis-disinserted blepharoptosis. Chronic tension-type headache sufferers were 3 to 15 times more likely than matched controls to receive a diagnosis of anxiety or mood disorder with almost half of the patients exhibiting clinically significant levels of anxiety and/or depression [85]. According to recent neuroimaging studies, one prominent theoretical perspective highlights the top–down inhibition of the amygdala by the ventromedial prefrontal cortex as a crucial neural mechanism that may be defective in particular mood and anxiety disorders [86]. These clinical findings support the hypothesis of functional connectivity among the mesencephalic trigeminal nucleus, locus coeruleus, and ventromedial prefrontal cortex.

## Conclusions

Eyelid opening with trigeminal proprioceptive activation may be able to both phasically and tonically regulate a physiological arousal mechanism in the brainstem [87]. Based on the neural connections among the trigeminal proprioceptive neurons, locus coeruleus, and ventromedial frontal cortex, we may wake by eyelid opening, rubbing the eyelids (mechanoreceptors in the supratarsal Müller muscle), or yawning with adequate trigeminal proprioceptive evocation. Memory retrieval could be evoked by upgaze (enhanced trigeminal proprioceptive evocation), meditating could be facilitated by downgazing (decreased trigeminal proprioceptive evocation), and non-rapid eye movement sleep could be induced by eyelid closing (lack of trigeminal proprioceptive evocation). Further studies are needed to confirm the connectivity using other methodologies and evaluate diseases that cause dysfunctions in the neural route by affecting Müller muscle mechanoreceptors, trigeminal proprioceptive neurons, the locus coeruleus, and the ventromedial prefrontal cortex.
